# Supplementary material for: DynOmics to identify delays and co-expression patterns across time course experiments
Source: Sci Rep. 2017 Jan 9;7:40131. doi: 10.1038/srep40131 (PMC5220332; doi:10.1038/srep40131)
Supplement: Supporting Information [file srep40131-s1.pdf]

A Supporting Material

Title: DynOmics to identify delays and co-expression patterns across time course experiments
Authors: [1]Jasmin Straube, [2+]Bevan Emma Huang, [3*+]Kim-Anh Lê Cao
Affiliations: [1]QFAB@QCIF Bioinformatics, Institute for Molecular Biosciences, The University of Queensland, Queensland Bioscience Precinct, St Lucia, QLD, Australia, [2]Janssen Research & Development, LLC, Discovery Sciences, Menlo Park, USA, [3]The University of Queensland Diamantina Institute, The University of Queensland, Translational Research Institute, Brisbane, QLD, Australia

A.1 Simulation study and method validation

Simulated data generation. We simulated data to evaluate and compare DynOmics to other methods to identify associations between trajectories. Data were generated based on similar scenarios to[1] with different parameters. Specifically, five reference levels were obtained using an impulse model and either 7 and 14 time points.[2] For each modelled reference, $P = 50$ queries with introduced time delay $-2, 1, 0, 1, 2$ (ten for each delay), were created. Moreover, we modelled $N = 50$ flat trajectories for each reference as negative control. Then different levels of normal distributed noise were added $\mathcal{N}(0, \sigma^2)$; $\sigma = 0.1, 0.2, 0.3, 0.5$. Each combination of varying number of time points and noise level was generated ten times. Table S1 presents an overview of the different parameters used for the simulated data. Figure S1 displays example references (black) and queries with different introduced noise $\sigma = 0.1, 0.5$, delays (color coded) and the random trajectories (grey).

Table S1. Simulated data schema. Presented is the number of time points, the number of different reference and query trajectories, the introduced delays, the number of flat trajectories, and the added noise per generated data set.

Time points	# of different expression trajectories	Delays for each trajectories	# of flat trajectories	Noise	# of generated datasets
7	5	-2, -1, 0, 1, 2 each repeated 10 times	50	0.1<br>0.2<br>0.3<br>0.5	10
14	5	-2, -1, 0, 1, 2 each repeated 10 times	50	0.1<br>0.2<br>0.3<br>0.5	10

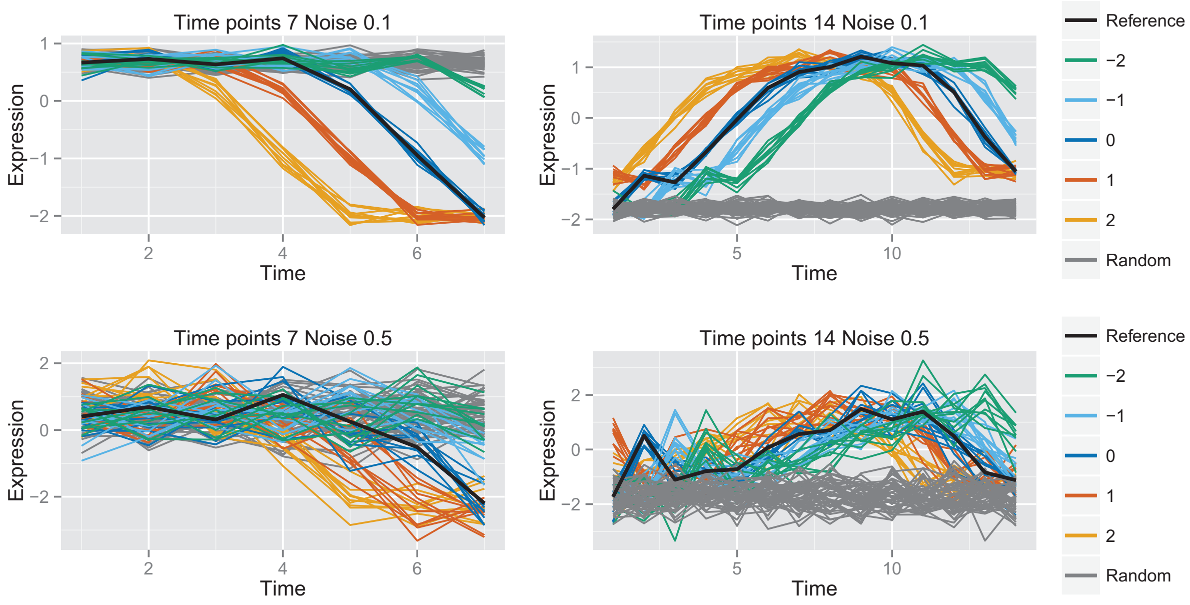

Figure S1. Examples of simulated trajectories. Presented are examples of simulated trajectories for 7 and 14 time points with different added normal distributed noise ($\sigma = 0.1, 0.5$). The reference (black) is displayed in concordance with the delayed query trajectories ranging from -2 to 2 coloured in green (-2), light blue (-1), dark blue (0), red (1), orange (2). The randomly generated expression trajectories are coloured in grey.

Fast Fourier transform visual explanation We describe the Fourier Transform components as depicted in Figure S2. For a given time series $x = (x_1, \dots, x_t, \dots x_T)$, $t = 1, \dots T$, let r denote its amplitude (size) and ϕ its phase angle (delay) of oscillations at different frequencies (speeds) k. The time series x is first decomposed into circular components for each frequency $k = 1, \dots, T-1$ as:

$$X_k = \frac{1}{T}\sum_{t=0}^{T-1} x_t e^{-i2\pi k\frac{t}{T}}. \tag{1}$$

As the amplitude at frequency $k = 0$ describes the y-axis offset (*i.e.* the global differences of expression levels), it is not included in our analysis context. Equation (1) can be written with polar coordinates with real part a and imaginary part b as $X_k = a_k + b_k i$ and represents the amount of frequency k in the time series (Figure S2 a). For each frequency $k = 1, \dots, T-1$ we can then calculate the amplitude r_k of the component, defined as $r_k = \sqrt{a_k^2 + b_k^2}$. The amplitude r_k (Figure S2 a; orange line) reflects the contribution of the frequency k to the overall pattern of the time series, and the maximum amplitude r_k describes the main pattern of the time series. In Figure S2 b we exemplify the deconstruction of a time series of length four into its oscillating components. The sum of the oscillations will then reconstruct the original time series.

The phase angle is the starting point on the circle that forms the pattern (Figure S2 a; grey point) and is usually defined as the argument of the FT at frequency k, denoted $Arg(X_k)$. For simplicity, we can transform the phase angle ϕ_k in degrees by:

$$\phi_k = \frac{180 * Arg(X_k)}{\pi}.$$

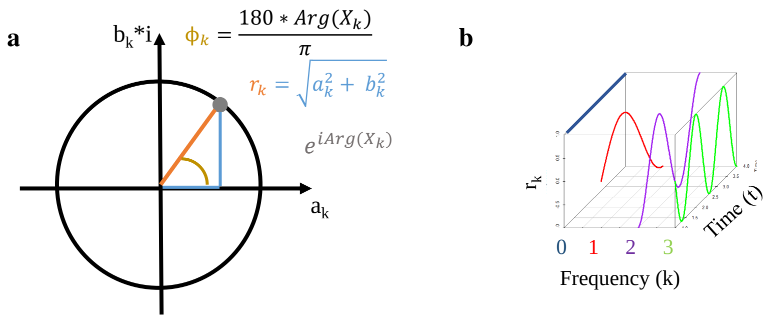

Figure S2. Diagram of Fourier transform components. **a**) represents the amplitude r of the circle and the phase angle ϕ for a given frequency. **b**) represents the decomposition of the signal for different frequencies k, and how many times the decomposed signature is 'spun' around the circle(*e.g.* once when k = 1, twice when k = 2, etc.) When $k = 0$, the amplitude represents the y-axis offset of the data (blue line).

Methods compared to DynOmics. We compared DynOmics with correlation methods namely (lagged) Pearson correlation and a Dynamic Time Warping (DTW) method DTW4Omics,[3] described briefly below.
Since the Pearson correlation cannot identify relationships between two trajectories if there is a time delay, we can overcome this issue by introducing time lags into x and y and choose the lag l that maximises the correlation between x and y. Let the lagged Pearson correlation be defined as in Equation 2 and the optimal delay as in Equation 3 (in the main article) the lags $\mathscr{L}$ for the lagged Pearson correlation is defined as $\mathscr{L} = \lfloor -\frac{T}{2} \rfloor, \cdots, \lfloor \frac{T}{2} \rfloor$. The trajectories were restricted to be lagged half of the trajectories' number of time points, since lagged Pearson correlation tended to maximise the delay to maximise correlation.
DTW4Omics obtains two trajectories and seeks for an alignment that minimizes the Euclidean distance (d) via inserting, deleting or matching the trajectories time points. The algorithm to minimize the distance between two trajectories x and y (DTWDist), with length T_x, T_y respectively, is defined as follows:

function DTWDIST(x,y)
  DTW = array $[0 \dots T_x, 0 \dots T_y]$
  for i = 1 to T_x **do** DTW[i, 0] = ∞
  end for ▷ initiation of infinite values to align each time point
  for i = 1 to T_y **do** DTW[0, i] = ∞
  end for
  DTW[0, 0] = 0
  for i = 1 to T_x **do**
    for j = 1 to T_y **do**

cost = d(x_i, y_j)
DTW[i, j] = cost + minimum(DTW[i-1, j], ▷ insertion
DTW[i , j-1], ▷ deletion
DTW[i-1, j-1]) ▷ match
end for
end for
return DTW[n, m]
end function

To determine if an alignment occurs by random chance a permutation test is used. The proposed test by Cavill *et al.*[3] independently permutes x_{perm} and y_{perm} then calculates the DTW distance as defined above,

$$Pvalue = \left(\sum_{k=1}^{100} (DTWDist(x_{perm}, y_{perm})_k < DTWDist(x,y)) \right) / 100. \quad (2)$$

As DTW4Omics generates P values as measurement of association, we also used the Pearson correlation of the DTW4Omics alignment (DTW4OmicsCor) for comparison. The estimated delay for DTW4Omics was the absolute maximum number of aligned time points to a single time point in either reference or query sequence.
A benchmark was created by realigning reference and query trajectories using the introduced delay and the Pearson correlation as a measurement of association. We referred to this benchmark as ‘Real delay’. We set a fixed threshold of 0.9 for correlation values and 0.05 for P values adjusted by false discovery rate (FDR).[4]

Sensitivity and specificity. The methods’ sensitivity and specificity were used to assess and compare the methods’ ability to identify associated reference-query trajectory pairs and disregard reference-random trajectory pairs on the simulated data. For each simulated data set we counted the number of true associated trajectories (true positives; TP), which was defined as a reference-query pair that is greater/smaller than the defined correlation ($cor > 0.9$) or P value threshold ($P < 0.05$). The number of true not associated or rejected trajectories, was a reference-random trajectory pair (true negative; TN) that was not greater or smaller than the defined correlation ($cor > 0.9$) or P value threshold ($P < 0.05$). We then calculated the sensitivity defined as the ratio of the TP and the number of truly associated trajectories (P) for each data set,

$$Sensitivity = \frac{TP}{P}. \quad (3)$$

The specificity was accordingly calculated for each simulated data set and was defined as the the ratio of the TN and the number of truly not-associated trajectories (N),

$$Specificity = \frac{TN}{N}. \quad (4)$$

Both measurements ranged between 0 and 1 with higher values indicating high sensitivity/specificity.

Results on simulated Data

To assess and compare DynOmics performance with current available methods we used the measures of sensitivity and specificity while identifying associations in simulated data. Generally, sensitivity performance decreased for all methods when noise increased. In terms of sensitivity DynOmics outperformed every method, when the number of time points was small (Figure S3 A; 7 time points). DynOmics’ average sensitivity ranged from 0.97 to 0.59. The next best performing algorithm was lagged Pearson correlation and DTW4OmicsCor, which identified at least 8% less true associated trajectories (sensitivity ranging from 0.89 to 0.51 and 0.82 to 0.46, respectively). The Pearson correlation that did not take time delays into account performed the worst with a sensitivity between 0.36 and 0.16 showing that ordinary correlation measurements is not sufficient to detect associations when trajectories are delayed. The specificity of DynOmics was slightly lower than the DTW methods (0.95-0.97) and Pearson correlation (1), however it was still greater than 0.94 which is a high specificity (Figure S3 B; 7 time points). The method with the lowest specificity was lagged Pearson correlation (0.91).

For a large number of time points sensitivity was extremely high ($1-0.95$) for all methods accounting for delays with noise $\sigma = \{0.1, 0.2, 0.3\}$ (Figure S3 A; 14 time points). The only method that does not account for delays, Pearson correlation, identified only 47 to 40% of all truly associated simulated trajectories. All methods performance dropped dramatically when the noise in the data increased ($\sigma = 0.5$). DTW4Omics the only method not based on Pearson correlation measurement was the only method that maintained high sensitivity (0.87) and was also better than the benchmark (0.6). Specificity was overall very high for all methods and noise levels ranging from 1 to 0.96 (Figure S3 B; 14 time points).
Furthermore, we investigated the methods’ ability to estimate the simulated time delay. DynOmics outperformed the other

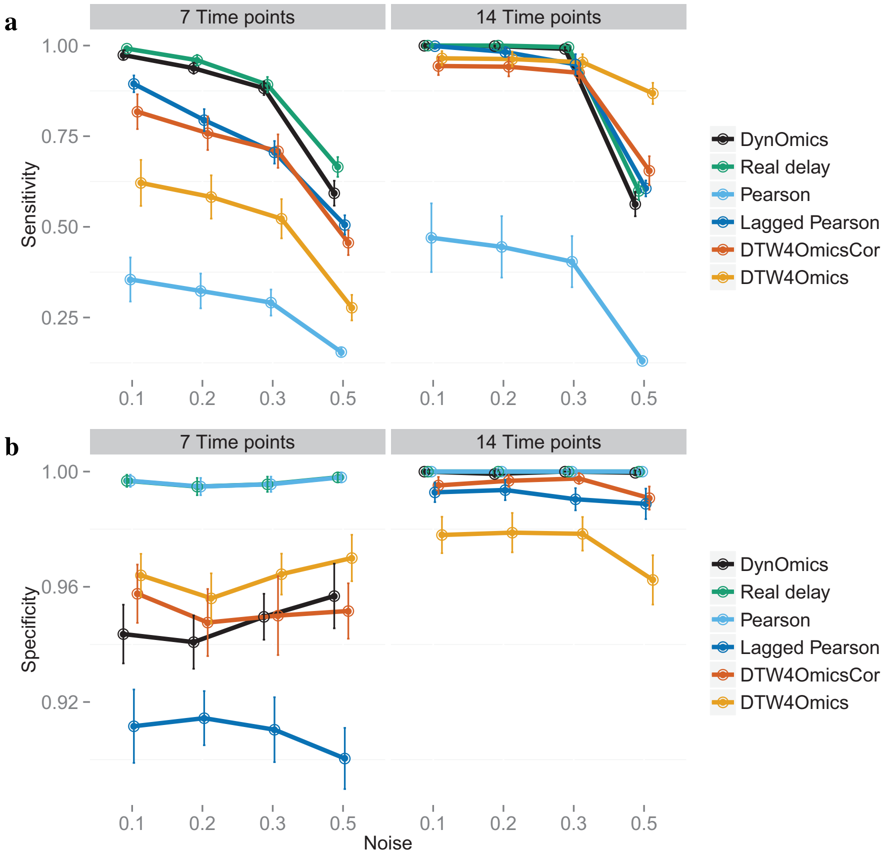

Figure S3. **Sensitivity and specificity on simulated data.** The graphs show each method's performance for 7 and 14 time points and different noises in terms **a**) sensitivity and **b**) specificity. The x-axis represents the variance of the introduced noise ($\sigma = \{0.1, 0.2, 0.3, 0.5\}$) of the analysed simulated data and the y-axis the mean (dot) and 95% confidence interval (error bar) of the respective performance measure. The proposed DynOmics approach (black) is visualized in comparison to the 'Real delay' used as benchmark (green), Pearson (light blue), lagged Pearson (dark blue), DTW4OmicsCor (red) and DTW4Omics (yellow).

methods in estimating the simulated delays for low number of time points (Table S2). Correct delay estimates for DynOmics ranged between 95 to 76%, while for lagged Pearson correlation and DTW4Omics they only ranged from 86 to 53% and 63 to 65%, respectively. For 14 time points DynOmics and lagged Pearson correlation performed similar with 99 to 90% and 100 to 94%, respectively, while DTW4Omics percentage of correct estimations was low ($60-42\%$). The separation of correct estimates by delay revealed that both, lagged Pearson correlation and DTW4Omics were inaccurate with increasing delay, while DynOmics estimates remained accurate for noise $\sigma = \{0.1, 0.2, 0.3\}$ (Figure S4).

Table S2. Percentage of correct estimated delays. The percentage of correct estimated delays is presented for DynOmics, lagged Pearson and DTW4Omics over all 500 generated associated trajectories per time point and noise combination.

		Percentage (%) correct estimated delays		
Time points	Noise	DynOmics	Lagged Pearson	DTW4Omics
7	0.1	95	86	63
	0.2	91	73	60
	0.3	85	61	58
	0.5	76	53	65
14	0.1	99	100	60
	0.2	98	97	48
	0.3	93	88	42
	0.5	90	94	53

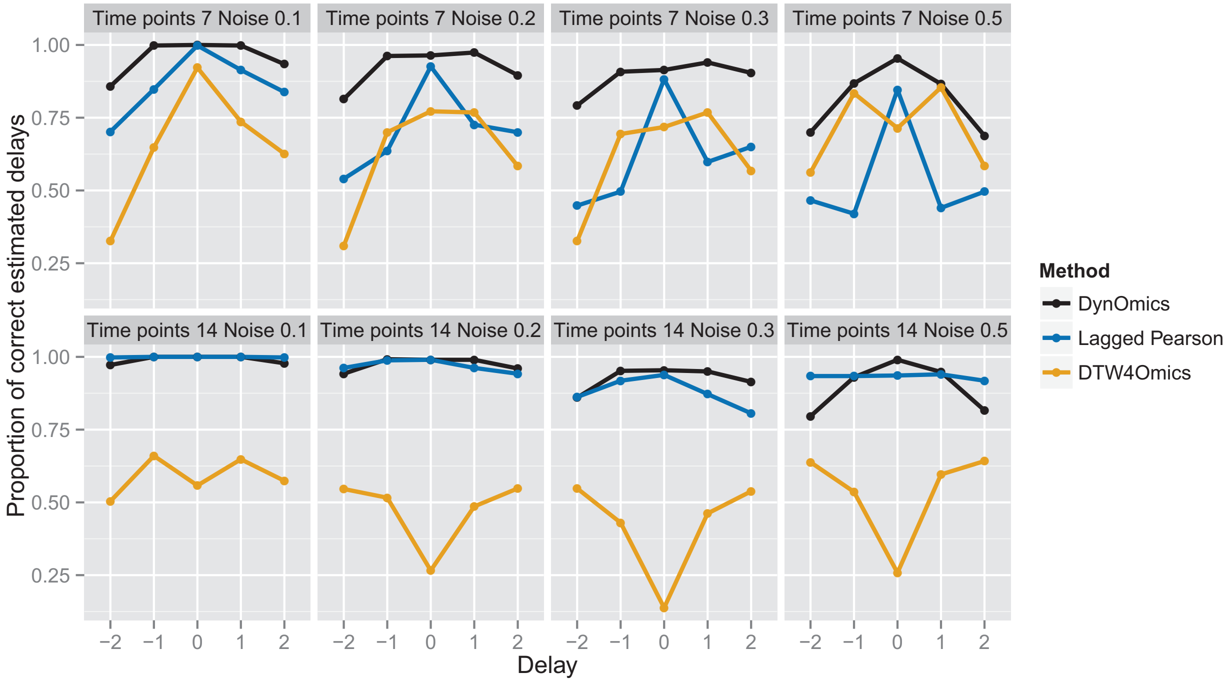

Figure S4. Proportion of correct estimated delays separated by simulated delay. For each combination of time points $(7,14)$ and noise ($\sigma = \{0.1, 0.2, 0.3, 0.5\}$) the proportion of the correct estimated delays is displayed on the y-axis (dot) against the originally simulated delay on the x-axis $(-2,-1,0,1,2)$. Colours represent the results for the different methods, namely DynOmics (black), Lagged Pearson (dark blue) and DTW4Omics (orange).

A.2 Lung Organogenesis

We compared the average overlap of identified association with correlation smaller than -0.9 between Pearson correlation of raw data ('Raw cor'), Pearson correlation on LMMS modelled data ('LMMS Cor') and DynOmics (Table S3). We observed that correlation analysis on the modelled data alone identified on average 85% more associated trajectories than correlation analysis on raw data. DynOmics had similar numbers as Pearson correlation on modelled data when comparing it to the correlation analysis on raw data, average of 86% more identified associated trajectories. There was a big overlap between DynOmics and Pearson correlation on modelled data suggesting that many associations had no or little time delay (82% and 86%). The on average 18% not identified my Pearson correlation on modelled data can be explained by time delay trajectories. The 14% of associations identified by Pearson correlation that were not identified by DynOmics were likely due to identifying optimal delays that resulted in positive correlations. These associations however, were not considered in this analysis, since we only compared negative correlated associations.

Table S3. **Average percentage of agreement and standard deviation (sd) of the identified associated mRNAs.** For each miRNA we calculated the percentage of agreement of the identified associations ($cor > 0.9$) using correlation on raw data ('Raw Cor'), on LMMS modelled ('LMMS Cor') and DynOmics. The numbers presented are the average agreement and the sd.

	Average Percentage % (sd)		
m	$\frac{RawCor \cap m}{\#RawCor}$	$\frac{LMMSCor \cap m}{\#LMMSCor}$	$\frac{DynOmics \cap m}{\#DynOmics}$
Raw Cor	-	15 (17)	14 (16)
LMMS Cor	79 (24)	-	82 (16)
DynOmics	77 (24)	86 (1)	-

We then compared the predicted miRNA targets based on co-expression and sequence similarity. Table S4-S7 show for each miRNA the number predicted targets based on co-expression for Pearson correlation on raw data and LMMS modelled data as well as DynOmics, and sequence similarity using microRNA.org, miRDB, and TargetScan, respectively. Figure S5 summarises the numbers for all miRNAs in boxplots. The figure suggestions that there is a big difference of number of predictions based on co-expression and sequence similarity depending on the method applied. For co-expression we observe that DynOmics and Pearson correlation on LMMS modelled data has most number of predictions and correlation analysis on raw data fewest (Figure S5 **a**). For predictions based on sequence similarity we observe that microRNA.org predicts on median around $\sim 3,000$ targets compared to miRDB and TargetScan which in median predict only ~ 400 (Figure S5 **b**). When comparing the percentage overlap of miRNA targets predicted by co-expression and sequence similarity (Figure S5 **c**) we observe little overlap. Overall DynOmics and Pearson correlation on LMMS modelled data have more overlap than Pearson correlation on raw data.

Table S4. Number of associated miRNA-mRNA expression trajectories. For each miRNA the overall number of identified associated mRNAs is presented when performing correlation analysis on the raw data (Raw Cor), LMMS modelled data (LMMS Cor) or DynOmics. DynOmics results are presented separated if mRNA expression changes after ($delay < 0$), simultaneous ($delay = 0$) or prior to ($delay > 0$) miRNA expression.

	Overall number of identified associations				
			DynOmics		
miRNA	Raw Cor	LMMS Cor	After	Simultanious	Prior
mmu-let-7c	1	3180	327	2572	379
mmu-let-7d	0	94	37	53	5
mmu-let-7e	2	64	8	55	15
mmu-let-7g	0	69	448	50	43
mmu-let-7i	0	109	35	38	7
mmu-miR-100	0	3180	336	2563	379
mmu-miR-106a	241	4338	761	3196	454
mmu-miR-126-3p	2140	3180	808	2058	412
mmu-miR-126-5p	1759	3180	418	2481	379
mmu-miR-127	44	168	25	123	26
mmu-miR-130a	0	4338	368	3622	421
mmu-miR-130b	140	4338	416	3499	496
mmu-miR-133a	362	3180	327	2572	379
mmu-miR-134	159	2347	556	2148	138
mmu-miR-135b	74	214	30	122	37
mmu-miR-136	7	65	8	33	6
mmu-miR-138	1	9	61	9	8
mmu-miR-139-5p	33	391	13	191	112
mmu-miR-140	3	3180	410	2489	379
mmu-miR-142-3p	908	3180	524	2375	379
mmu-miR-145	7	3180	334	2564	380
mmu-miR-146a	1967	3476	475	1184	2007
mmu-miR-146b	1741	3013	286	1100	1699
mmu-miR-149	0	4338	530	3292	589
mmu-miR-150	2384	3180	327	2572	379
mmu-miR-151-3p	4	3180	362	2504	412
mmu-miR-15a	0	3180	332	2567	379
mmu-miR-15b*	0	4338	494	3496	421
mmu-miR-16	564	3180	451	2445	382
mmu-miR-17	142	4338	583	3358	470
mmu-miR-181a	2	3180	337	2562	379
mmu-miR-182	37	4338	566	3373	472
mmu-miR-191	276	3180	327	2572	379
mmu-miR-195	850	3180	382	2475	421
mmu-miR-19a	227	2468	319	2238	169
mmu-miR-19b	163	4338	487	3503	421
mmu-miR-200a	687	1719	642	1123	148
mmu-miR-200b	40	542	49	496	19
mmu-miR-200c	37	294	19	277	12
mmu-miR-20a	197	4338	367	3623	421
mmu-miR-21	1407	3180	332	2567	379
mmu-miR-210	4	52	0	38	14
mmu-miR-214	14	4338	607	3382	422
mmu-miR-214*	202	4338	367	3623	421
mmu-miR-222	1579	3180	390	2445	443
mmu-miR-223	2086	3537	479	3035	146
mmu-miR-24	1181	3180	340	2559	379
mmu-miR-24-2*	752	3180	832	2034	412
mmu-miR-26a	326	3180	369	2530	379
mmu-miR-26b	770	3180	327	2572	379

Table S4. **Number of associated miRNA-mRNA expression trajectories.**

mmu-miR-27a	429	3180	328	2571	379
mmu-miR-27b	0	3180	327	2572	379
mmu-miR-28*	0	3180	343	2556	379
mmu-miR-296-5p	21	232	33	166	17
mmu-miR-29a	2006	3180	328	2571	379
mmu-miR-29c	964	2713	2209	535	227
mmu-miR-301a	10	4338	395	3595	421
mmu-miR-301b	25	284	43	197	42
mmu-miR-30a	1529	3180	328	2571	379
mmu-miR-30a*	642	3180	462	2429	387
mmu-miR-30b	138	3180	327	2572	379
mmu-miR-30c	559	3180	446	2423	409
mmu-miR-30d	2021	3180	468	2338	472
mmu-miR-30e	1491	3180	481	2204	593
mmu-miR-30e*	608	3180	328	2571	379
mmu-miR-31	348	3180	635	2255	388
mmu-miR-322	54	219	3	131	13
mmu-miR-322*	5	53	4	39	1
mmu-miR-323-3p	92	4338	927	3054	430
mmu-miR-328	19	3180	334	2565	379
mmu-miR-335-3p	22	84	11	50	4
mmu-miR-335-5p	1	55	2	37	4
mmu-miR-34b-3p	2159	3180	514	2327	437
mmu-miR-351	0	94	5	70	10
mmu-miR-365	529	3180	665	2234	379
mmu-miR-370	13	4338	710	3117	584
mmu-miR-375	0	3180	543	2356	379
mmu-miR-376c	53	190	24	57	32
mmu-miR-379	74	207	23	111	26
mmu-miR-382	12	272	56	214	25
mmu-miR-409-3p	173	295	257	182	91
mmu-miR-410	124	312	159	199	81
mmu-miR-411	41	163	20	107	11
mmu-miR-429	99	226	2670	197	3
mmu-miR-431	97	255	141	198	33
mmu-miR-433	12	4338	380	3610	421
mmu-miR-434-3p	13	85	9	29	14
mmu-miR-449a	147	444	30	214	18
mmu-miR-466d-3p	680	3180	484	2376	418
mmu-miR-467a*	39	433	230	205	238
mmu-miR-486	926	3180	332	2567	379
mmu-miR-503	9	73	4	64	10
mmu-miR-503*	18	224	25	128	20
mmu-miR-532-5p	1	4338	849	2978	584
mmu-miR-539	92	250	148	196	35
mmu-miR-672	21	130	23	69	42
mmu-miR-680	0	3180	368	2531	379
mmu-miR-690	0	4338	737	3253	421
mmu-miR-699	0	4338	376	3614	421
mmu-miR-708	12	80	270	73	9
mmu-miR-709	0	4338	367	3623	421
mmu-miR-805	0	4338	520	3470	421
mmu-miR-877*	0	3180	743	2064	471
mmu-miR-92a	62	4338	766	3188	457

Table S5. **Overlap of predicted miRNA targets from microRNA.org and associated miRNA-mRNA expression trajectories.** Presented are the number of miRNA target predictions in the microRNA.org database (DB) (No. DB prediction), the number of associated miRNA-mRNA trajectories that overlap with the mircoRNA.org DB predictions for correlation analysis on the raw data (Raw Cor) and LMMS modelled data (LMMS Cor). Finally, the number of overlaps between the miRNAs targets of microRNA.org and DynOmics are presented separated if mRNA expression changes after ($delay < 0$), simultaneous ($delay = 0$) or prior to ($delay > 0$) miRNA expression.

		microRNA.org overlap				
				DynOmics		
miRNA	No. DB prediction	Raw Cor	LMMS Cor	After	Simultanious	Prior
mmu-let-7c	2659	0	49	4	39	7
mmu-let-7d	2610	0	3	0	3	0
mmu-let-7e	2673	0	1	0	1	0
mmu-let-7g	2596	0	2	8	1	1
mmu-let-7i	2558	0	3	0	0	0
mmu-miR-100	373	0	5	0	5	0
mmu-miR-106a	3621	4	79	14	65	7
mmu-miR-126-3p	87	1	3	1	2	0
mmu-miR-127	420	0	0	0	0	0
mmu-miR-130a	2887	0	66	3	58	9
mmu-miR-130b	2962	4	65	2	56	11
mmu-miR-133a	1780	2	49	1	44	8
mmu-miR-134	2632	4	31	9	26	4
mmu-miR-135b	3433	2	4	1	1	3
mmu-miR-136	3355	0	1	0	0	0
mmu-miR-138	2933	0	0	0	0	0
mmu-miR-139-5p	3154	1	10	0	6	3
mmu-miR-140	2416	0	64	7	52	7
mmu-miR-142-3p	2060	10	47	10	33	5
mmu-miR-145	3220	0	54	5	47	4
mmu-miR-146a	3334	31	55	6	24	32
mmu-miR-146b	3323	30	45	6	18	23
mmu-miR-149	3332	0	74	4	61	12
mmu-miR-150	2643	47	63	10	47	11
mmu-miR-15a	4302	0	88	11	73	8
mmu-miR-15b*	4394	0	97	14	78	9
mmu-miR-16	4232	18	84	13	67	8
mmu-miR-17	3695	5	79	9	68	7
mmu-miR-181a	4517	0	105	12	85	11
mmu-miR-182	3235	0	65	7	54	5
mmu-miR-191	932	2	23	1	21	3
mmu-miR-195	4116	19	81	12	64	9
mmu-miR-19a	3312	3	33	7	28	3
mmu-miR-19b	3195	3	68	8	53	10
mmu-miR-200a	4222	19	46	17	32	4
mmu-miR-200b	3808	2	15	2	14	0
mmu-miR-200c	3794	1	6	0	6	0
mmu-miR-20a	3803	3	78	6	69	8
mmu-miR-21	2340	29	47	4	41	5
mmu-miR-210	839	0	0	0	0	0
mmu-miR-214	3970	0	73	5	60	12
mmu-miR-214*	6310	8	122	9	103	16
mmu-miR-222	2541	26	48	9	37	4
mmu-miR-223	2518	38	58	13	45	4
mmu-miR-24	3371	20	55	6	47	6
mmu-miR-26a	3287	10	69	8	58	6
mmu-miR-26b	3388	15	69	8	58	7

Table S5. **Overlap of predicted miRNA targets from miRNA.org and associated miRNA-mRNA expression trajectories.**

mmu-miR-27a	4060	14	87	13	71	5
mmu-miR-27b	4131	0	84	12	70	4
mmu-miR-28*	2657	0	52	1	44	11
mmu-miR-29a	2871	27	46	3	39	8
mmu-miR-29c	2918	16	38	28	9	2
mmu-miR-301a	3271	0	81	6	69	11
mmu-miR-301b	3238	0	6	0	6	1
mmu-miR-30a	4250	43	74	9	58	14
mmu-miR-30a*	4250	14	74	11	57	14
mmu-miR-30b	4288	8	78	8	62	14
mmu-miR-30c	4350	14	78	11	62	11
mmu-miR-30d	4079	52	72	12	54	13
mmu-miR-30e	4395	44	75	10	57	17
mmu-miR-30e*	4395	12	75	9	59	15
mmu-miR-31	2985	11	66	10	47	15
mmu-miR-322	4334	2	6	0	3	1
mmu-miR-322*	6924	0	2	0	1	0
mmu-miR-328	1864	1	33	1	28	5
mmu-miR-335-5p	3120	0	1	1	0	0
mmu-miR-351	2210	0	1	0	1	0
mmu-miR-365	1561	13	42	5	34	4
mmu-miR-370	2978	0	55	11	39	8
mmu-miR-375	1006	0	22	10	14	0
mmu-miR-376c	3544	3	5	0	3	0
mmu-miR-379	1493	0	2	0	0	1
mmu-miR-382	3174	1	7	0	5	0
mmu-miR-410	4653	4	9	2	4	4
mmu-miR-411	1741	1	2	0	1	0
mmu-miR-429	3891	3	6	73	4	0
mmu-miR-431	2037	0	0	3	0	0
mmu-miR-433	2651	0	67	10	52	8
mmu-miR-449a	3163	2	11	0	4	1
mmu-miR-486	1750	4	27	3	22	3
mmu-miR-503	984	0	0	0	0	0
mmu-miR-503*	984	0	0	0	0	0
mmu-miR-539	5055	2	9	2	7	2
mmu-miR-708	2757	0	0	6	0	0
mmu-miR-92a	2168	1	36	9	25	3

Table S6. Overlap of predicted miRNA targets from miRDB associated miRNA-mRNA expression trajectories. Presented are the number of miRNA target predictions in the miRDB database (DB) (No. DB prediction), the number of associated miRNA-mRNA trajectories that overlap with the miRDB predictions for correlation analysis on the raw data (Raw Cor) and LMMS modelled data (LMMS Cor). Finally, the number of overlaps between the miRNAs targets of miRDB and DynOmics are presented separated if mRNA expression changes after ($delay < 0$), simultaneous ($delay = 0$) or prior to ($delay > 0$) miRNA expression.

		miRDB overlap				
				DynOmics		
miRNA	No. DB prediction	Raw Cor	LMMS Cor	After	Simultanious	Prior
mmu-miR-139-5p	428	0	1	0	1	0
mmu-miR-151-3p	134	0	4	0	4	0
mmu-miR-296-5p	462	0	0	0	0	0
mmu-miR-323-3p	410	0	8	1	7	0
mmu-miR-335-3p	1190	1	1	0	1	0
mmu-miR-335-5p	410	0	0	0	0	0
mmu-miR-34b-3p	245	1	2	0	2	0
mmu-miR-409-3p	291	0	1	2	0	0
mmu-miR-434-3p	173	0	0	0	0	0
mmu-miR-466d-3p	1265	4	23	3	17	3
mmu-miR-532-5p	279	0	4	1	3	0
mmu-miR-680	341	0	8	4	4	1
mmu-miR-690	319	0	8	1	6	1
mmu-miR-709	1494	0	19	1	18	0

Table S7. Overlap of predicted miRNA targets from TargetScan associated miRNA-mRNA expression trajectories. For each miRNA the number of miRNA target predictions in the TargetScan database (DB) (No. DB prediction) and the overlap with the expression data analysis are presented. The number of associated miRNA-mRNA trajectories that overlap with the TargetScan DB predictions for correlation analysis on the raw data (Raw Cor) and LMMS modelled data (LMMS Cor). Finally, the number of overlaps between the miRNAs targets of TargetScan and DynOmics are presented separated if mRNA expression changes after ($delay < 0$), simultaneous ($delay = 0$) or prior to ($delay > 0$) miRNA expression.

		TargetScan overlap				
				DynOmics		
miRNA	No. DB prediction	Raw Cor	LMMS Cor	After	Simultanious	Prior
mmu-miR-126-3p	25	0	0	0	0	0
mmu-miR-127	21	1	1	0	1	0
mmu-miR-134	249	0	6	2	3	2
mmu-miR-136	369	1	1	0	1	0
mmu-miR-138	773	0	0	0	0	0
mmu-miR-139-5p	503	0	1	0	1	0
mmu-miR-140	461	0	15	1	12	3
mmu-miR-142-3p	481	4	16	3	12	1
mmu-miR-145	928	0	20	1	17	2
mmu-miR-149	550	0	14	1	11	2
mmu-miR-150	379	5	7	2	5	0
mmu-miR-17	1563	2	28	4	24	4
mmu-miR-182	1471	0	25	5	18	3
mmu-miR-191	98	0	2	0	2	0
mmu-miR-21	421	5	18	0	17	2
mmu-miR-210	40	0	0	0	0	0
mmu-miR-214	895	0	18	1	16	1
mmu-miR-214*	1316	1	22	1	20	1
mmu-miR-223	466	3	7	0	7	0
mmu-miR-24	824	7	19	3	16	1
mmu-miR-31	530	2	13	1	12	2
mmu-miR-370	547	0	14	3	11	2
mmu-miR-375	292	0	8	2	6	1
mmu-miR-376c	396	0	1	0	0	0
mmu-miR-379	133	0	0	0	0	0
mmu-miR-382	293	0	0	0	0	0
mmu-miR-410	856	0	1	0	1	0
mmu-miR-411	120	0	0	0	0	0
mmu-miR-431	227	0	0	0	0	0
mmu-miR-433	496	0	17	3	12	3
mmu-miR-503	477	0	0	0	0	0
mmu-miR-503*	477	0	1	0	0	0
mmu-miR-539	936	1	1	2	1	0

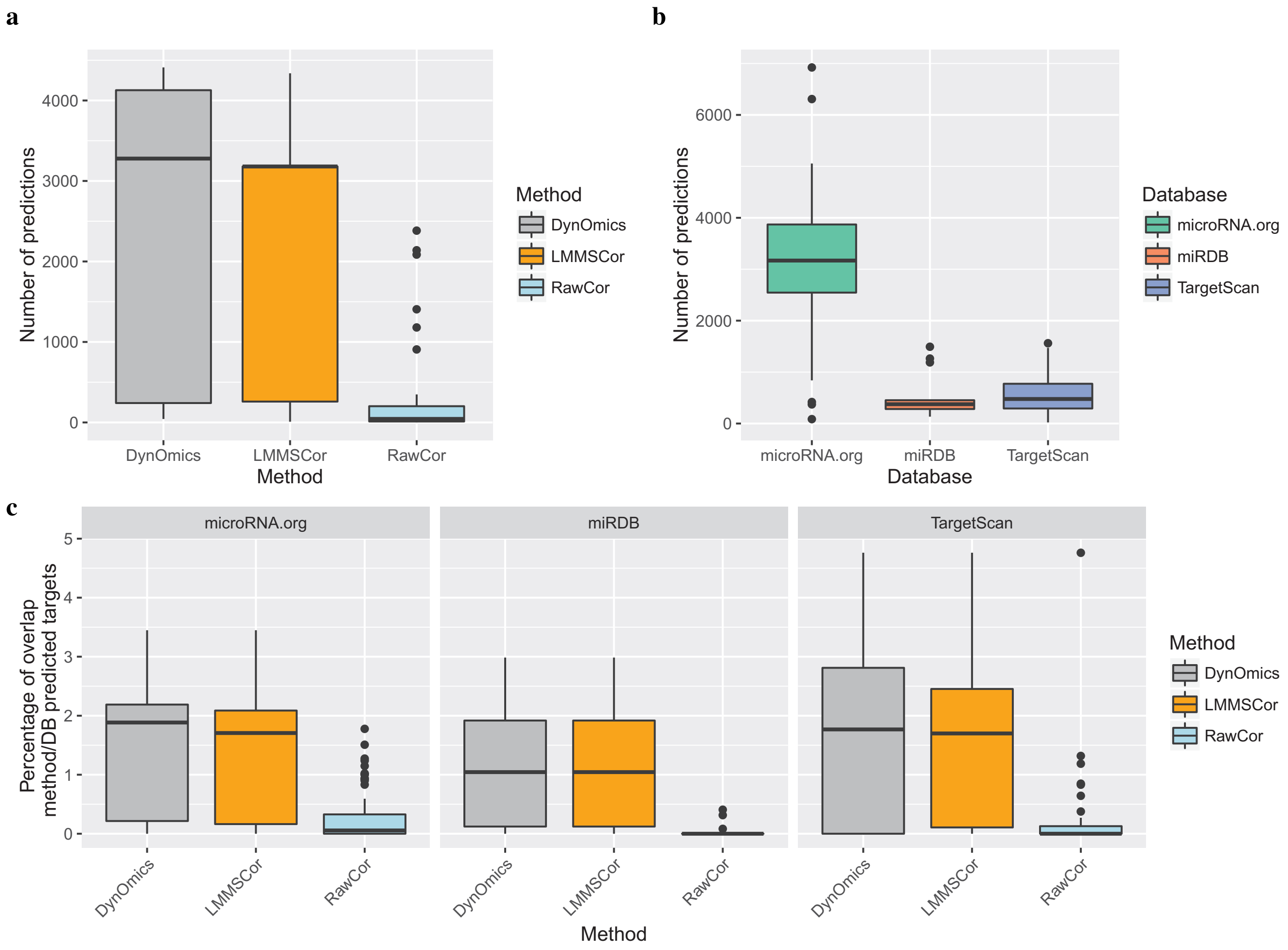

Figure S5. **Summary of miRNA predictions based on co-expression and sequence similarity** Presented is a visual summary of Supporting Tables S4-S7. In **a**) we present summarised as boxplots the number of putative miRNA targets based on co-expression (cor < -0.9) for DynOmics (grey), Pearson correlation on LMMS modelled data (orange) and on raw data (lightblue). In **b**) we depict the number of predictions of putative miRNA targets for databases microRNA.org (green), miRDB (red) and TargetScan (purple). In **c**) for each database we present the percentage of overlap of predicted putative miRNA targets based on sequence similarity with the predictions made based on co-expression.

References

1. Redestig, H. & Costa, I. G. Detection and interpretation of metabolite-transcript coresponses using combined profiling data. *Bioinformatics* **27**, i357–65 (2011).
2. Chechik, G. & Koller, D. Timing of gene expression responses to environmental changes. *J. Comp. Biol* **16**, 279–290 (2009).
3. Cavill, R., Kleinjans, J. & Briede, J.-J. DTW4Omics : Comparing Patterns in Biological Time Series. *PLOSONE* **8**, e71823 (2013).
4. Benjamini, Y. & Hochberg, Y. Controlling the false discovery rate: A practical and powerful approach to multiple testing. *J. R. Stat. Soc. Ser. B Stat. Soc.* **57**, 289–300 (1995).
